# HBV reactivation in an occult HBV infection patient treated with prednisone for nephrotic syndrome: case report and literature review

**DOI:** 10.1186/1471-2334-13-394

**Published:** 2013-08-27

**Authors:** Wenjun Du, Zhaomin Zheng, Shaolei Han, Shumin Ma, Shijun Chen

**Affiliations:** 1 Department of Liver Disease, Jinan Infectious Disease Hospital, Shandong University School of Medicine, 22029# Jing-Shi Road, Jinan 250021, China

**Keywords:** Hepatitis B virus reactivation, IgM nephropathy, Occult HBV infection, Prednisone

## Abstract

**Background:**

Reactivation of hepatitis B virus (HBV), characterized by increased levels of serum HBV DNA, abnormal liver function and hepatic failure, is a frequent complication of immunosuppressive therapy and chemotherapy in patients with HBV infection. However, reactivation of occult HBV infection with immunosuppressive therapy or chemotherapy is rare.

**Case presentation:**

A 77-year-old man was diagnosed with nephrotic syndrome and IgM nephropathy with unclear pathogenesis. Liver function was normal, HBV-related serum markers were negative and HBV DNA titer was below the upper limits of normal. Two months following the start of prednisone therapy for his nephrotic syndrome, laboratory tests revealed a substantial increase in serum transaminase levels (ALT: 490 IU/L; AST: 149 IU/L) and an elevation of HBV DNA level (3.42×10^6^ copies/ml). We tested stored kidney tissue for HBsAg and HBcAg using immunohistochemistry and found the sample to be HBcAg positive, allowing us to confirm the etiology of nephropathy as an occult HBV infection. The cause of the hepatitis was thought to be HBV reactivation, so we immediately administered lamivudine. One month after the initiation of daily lamivudine treatment, laboratory tests revealed that serum levels of transaminases had improved (ALT: 35 IU/L; AST: 17 IU/L). Patient examination one year later showed that HBeAg had decreased with a concomitant increase of HBeAb, the quantity of HBV DNA was undetectable, and liver function and renal function had stabilized.

**Conclusion:**

This is the first report describing HBV reactivation in an occult HBV infection patient treated with oral prednisone for nephrotic syndrome. HBV-associated antigen should be regularly tested for in patients with unknown etiological glomerulonephritis in areas with high HBV viral popular and even in those with no clinical evidence for diagnosis of HBV.

## Background

Hepatitis B virus (HBV) infection has been shown to induce extrahepatic lesions, commonly through the deposition of immune complexes, in different organs. HBV-associated glomerulonephritis (HBVAN) is one of the more important extrahepatic diseases, and nephrotic syndrome is its most common clinical manifestation [1-8]. Pathological types of HBVAN include membranous nephropathy, minimal change nephropathy, mesangial proliferative glomerulonephritis, membranoproliferative glomerulonephritis and IgA nephropathy. Of these, membranous nephropathy is the most common, and reports of IgM nephropathy are rare.

Occult HBV infection is defined as the undetectable of HBV DNA and the absence of HBV surface antigen in plasma or serum of HBV-infected patients [1,9]. This infection may persist in individuals for years before any symptoms of overt HBV infection emerge. Previous studies reported that co-infection [10], drug abuse [11] and immunosuppression [12] can trigger an increase of HBV DNA levels without an increase of hepatitis B s antigen (HBsAg).

This is the first report describing HBV reactivation in an occult HBV infection patient treated with oral prednisone for nephrotic syndrome. HBV reactivation allowed for definitive characterization of the pathogenesis of nephrotic syndrome in this particular case, which has been difficult in past cases.

## Case presentation

**Figure 1:**
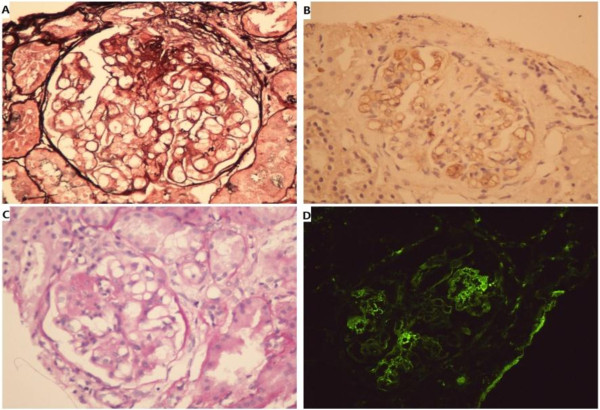
**Image of renal biospy. A**: Focal segmental glomerulosclerosis with glomerular capillary wall collapse, balloon adhesion and fibrous small new moon body form. Surrounding the open capillary lumen, the mesangial area has no obvious proliferation. (silver staining, 400x). **B**: Segmental glomerular sclerosis, capillary bundle segmental collapse, balloon adhesion and cell sex small new moon body form. (PAS staining, 400x). **C**: HBcAg immunohistochemistry staining: HBcAg along the glomerular capillary wall and mesangial area; stage positive. (400x). **D**: Immunofluorescence: The glomerulus and mesangial area along the grain; sample fluorescence distribution: LgG-, LgA-, LgM++, C3-, F-, C_1_q-.

A 77-year-old man was diagnosed with nephrotic syndrome with laboratory tests showing urine protein of ++++, urinary occult blood test of ++++, 24-hour urine total protein of 9.99 g/L, serum albumin of 25.2 g/L, creatinine (Cr) of 234 μmol/L and blood urea nitrogen (BUN) of 13.5 mmol/L. Detection of HBV-related serum makers by chemiluminescent immunoassay showed that HBsAg, anti-HBs, hepatitis B e antigen (HBeAg), anti-HBe and anti-HBc were all negative. Furthermore, the HBV DNA titer was undetectable (<10^3^ copies/ml). Anti-hepatitis C virus (HCV) was negative, anti-hepatitis E virus (HEV) was negative, and both alanine aminotransferase (ALT) and aspartate aminotransferase (AST) were normal (below 40 IU/L). To further confirm the pathogenesis of nephrotic syndrome, the patient received a renal biopsy. As shown in (Figure 1A,B,D), the results showed IgM nephropathy (focal segmental glomerulosclerosis and crescent formation) and diffuse damage to the renal tubules and stroma. The pathological diagnosis was nephrotic syndrome with IgM nephropathy; however, the exact cause of nephrosis was unclear. At that time, the patient was being administered prednisone at a dose of 50 mg once daily.

Two months after starting treatment, laboratory tests revealed a substantial increase in serum transaminase levels (AST: 149 IU/L; ALT: 490 IU/L), urine protein of ++, urinary occult blood test of +++, 24-hour urine total protein of 3.1 g/L, serum albumin of 28.1 g/L, Cr of 211 μmol/L and BUN of 19.3 mmol/L. Strangely, HBV markers were now positive for HBsAg, HBcAb and HBeAg, and the quantity of HBV DNA had increased to 3.42×10^6^ copies/ml. Anti-HCV and anti-HEV were still negative, and autoimmune hepatitis indices, including anti-nuclear antibody (ANA), anti-mitochondrial antibody (AMA) and liver kidney microsomal antibody (LKM), and serum markers related to other hepatitis infections, such as hepatitis A virus (HAV), cytomegalovirus, Epstein-Barr virus and herpes virus, were all negative. Ultrasound of the liver showed chronic hepatic injury.

Pathological diagnosis using immunohistochemistry to detect the existence of S-Ag or C-Ag in renal tissue is currently regarded as the “gold standard” for diagnosis of HBVAN. We therefore tested stored kidney tissue for HBsAg and HBcAg using immunohistochemistry and found the sample to be HBcAg positive (Figure 1C). In addition, molecular analysis showed that the HBV genotype was C with no HBV mutation in the pre-core or core promoter regions, thus we concluded that the cause of the acute hepatitis was HBV reactivation, and the pathogenesis of nephrotic syndrome was attributed to HBVAN. We immediately administered lamivudine at a dose of 100 mg once daily. One month after starting the daily lamivudine treatment, laboratory tests revealed that the serum levels of aminotransferase (ALT) and aspartate aminotransferase (AST) had improved (ALT: 35 U/L, AST: 17 U/L), the quantity of HBV DNA was 1.1×10^5^ copies/ml, BUN was 11.3 mmol/L and Cr was 119 μmol/L. One year after starting the treatment for HBV reactivation, follow-up tests showed that HBsAg was positive, HBsAb was negative, HBeAg was negative, HBeAb was positive, HBcAb was positive, BUN was 10.8 mmol/L, Cr was 121 μmol/L and HBV DNA was undetectable by real-time PCR.

### Discussion

Occult HBV infection is defined as infection with detectable HBV DNA and surface antigen (HBsAg) in the blood [9]. This infection may persist in individuals for years before any symptoms of overt HBV infection emerge, and the causes associated with the transition from an overt HBV infection to an occult infection are currently unknown.

HBVAN is an extrahepatic manifestation of HBV infection, and its main clinical manifestation is nephrotic syndrome, which predominantly occurs during childhood and in males [1-6]; however, the number of reports on adult patients is very limited [7,8]. Data suggests that, compared to adults, the prognosis of HBVAN is more favorable in children and progression to renal failure is more rare [6,8]. Pathological types of HBVAN include membranous nephropathy, minimal change nephropathy, mesangial proliferative glomerulonephritis, membranoproliferative glomerulonephritis and IgA nephropathy. Of these, membranous nephropathy is the most common, and reports of IgM nephropathy are rare [1-8]. IgM nephropathy, a minimal change nephropathy rarely associated with HBVAN, is defined by the presence of diffuse and global mesangial deposits of IgM [13]. Many glomerulopathies are accompanied by IgM deposition, so some experts have suggested the classification of IgM nephropathy as its own subtype of minimal change nephropathy and an independent diagnosis of IgM nephropathy past a certain level of deposition intensity. The pathogenesis of IgM nephropathy is currently unclear, but the accepted view of some authorities is that immune complex deposition may cause injury to the glomerular filtration membrane, and the deposited antigen may originate from microbes, drugs or heavy metals [13]. In our case, HBV antigen complex deposition in the glomerular filtration membrane may have led to the observed injury.

A ‘full house’ (IgG, IgA, IgM, C3 and C1q) positive immunoflurescence staining is typically included in the pathological diagnosis of HBVGN. The patient in this case report was misdiagnosed due to the absence of ‘full house’ positive staining, instead having only IgM-positive staining, and lack of positive clinical proof of HBV-associated markers in the serum. Given the presence of IgM, it is possible that, during the early stages of HBV development, the low HBV DNA load and low levels of other related antigens allowed for the specific deposition of IgM in the glomerular filtration membrane. Increasing diagnostic experience suggests that positive antigen or visible viral particles in the tissue are more important than previously thought for diagnosis. These patients could potentially exhibit a positive serum test upon subsequent follow-up.

Nephrotic syndrome was the original clinical manifestation of our patient, and a renal biopsy was taken to confirm pathology. Chronic HBV was not recognized, as HBV-related serum markers were negative and HBV DNA was below the upper limits of normal, so HBV-related antigen detection with immunohistochemistry was subsequently ignored. Further analysis of the biopsy results revealed IgM nephropathy, of which previous reports are few. The patient was administered the immunosuppressive drug prednisone to treat the nephrotic syndrome without preemptive therapy with antiviral drugs. Two months later, elevation of ALT and AST and detectable HBV DNA suggested a hepatitis B flare, which was likely due to the prolonged duration of immunosuppressive therapy resulting in HBV reactivation.

The HBV genotype influences clinical outcomes, serum HBV DNA levels and mutational patterns in the pre-core and core promoter regions [14]. For this particular patient, the HBV genotype was C, but serum HBV DNA levels at the time we initiated treatment with prednisone were low, and no HBV mutations were observed in the pre-core or core promoter regions. These findings lead us to surmise that HBV genotype directly influences HBV reactivation associated with immunotherapy. The inhibition of prednisone may be relevant to the replication of HBV, though the specific mechanism remains unclear.

Preemptive therapy with lamivudine for HBsAg-positive patients undergoing chemotherapy reduces the risk of HBV reactivation and HBV-associated morbidity and mortality [15]. Meanwhile, improvements in liver disease and renal function have been reported following clearance of HBsAg [16]. We therefore treated the patient with lamivudine to repress the replication of HBV DNA, expecting subsequent improvement of renal function. One month after initiating lamivudine treatment, liver function was improved and renal function had not worsened. The patients remained on lamivudine (100 mg/day) therapy for one year. Follow-up tests showed that HBsAg was positive, HBsAb was negative, HBeAg was negative, HBeAb was positive, HBcAb was positive, BUN was 10.8 mmol/L, Cr was 121 μmol/L, urine protein of +, urinary occult blood test of ++, 24-hour urine total protein of 2.14 g/L, serum albumin of 30.2 g/L, and HBV DNA was undetectable by real-time PCR. The main indeces for liver function include ALT, AST and total bilirubin (TBIL) were all below the upper limits of normal. Total cholesterol (TC) and thyroglobulin (Tg) levels were 7.4 mmol/L and 2.5 mmol/L, respectively.

## Conclusion

In conclusion, HBV-associated antigen should be regularly tested for in patients with unknown etiological glomerulonephritis in areas with high HBV viral popular and even in those with no clinical evidence for diagnosis of HBV. IgM nephropathy as a pathological diagnosis should not be ignored in cases of HBVAN, especially in older patients. Following a definite diagnosis, if a patient with HBV infection is to be treated with immunosuppressive agents, preemptive therapy with antiviral agents is recommended for its contribution to decreasing HBV reactivation and hepatitis B flare.

### Consent

Written informed consent was obtained from the patient for publication of this case report and any accompanying images. A copy of the written consent is available for review by the Editor of this journal.

## Competing interest

The authors declare that they have no potential conflicts of interest to disclose.

## Authors’ contributions

Study concept and design: CSJ. Acquisition of data: DWJ and ZZM. Analysis and interpretation of data: HSL and MSM. Drafting of the manuscript: DWJ and MSM. Critical revision of the manuscript for important intellectual content: CSJ. All authors read and approved the final manuscript.

## Pre-publication history

The pre-publication history for this paper can be accessed here:

http://www.biomedcentral.com/1471-2334/13/394/prepub
